# Supplementary material for: Learning curve and surgical outcome of robotic assisted colorectal surgery with ERAS program
Source: Sci Rep. 2022 Nov 29;12:20566. doi: 10.1038/s41598-022-24665-w (PMC9709162; doi:10.1038/s41598-022-24665-w)
Supplement: Supplementary file 1 — Supplementary Information 1. [file 41598_2022_24665_MOESM1_ESM.zip › LSC Raw data-Ñ[▒K/066/H_ñw╝╨░O▒Kñσ.pdf]

正式摘要(電子簽章：000000185808697)<br>W83 / W83 ☐英文標題(注意:無英文姓名-請補病人基本檔!)	☐【讀取】憑證	

臺中榮民總醫院
出院病歷摘要

轉入號： 轉出號：

[redacted]

(8)流水編號	
(9)入院日期	109/02/06 (2020) 直腸外科 W83 - 096
(10)轉科日期	
(11)出院日期	109/02/14 (2020) 住院天數計 8 日

診斷

- 入院:Ca of Descending colon
- 出院:Ca of Descending colon s/p Lapa AR on 2020/02/07

主訴

- abnormal tumor marker elevation during GS follow up

病史

- This is a 50 year old female patient with history of
  1. Ca of breast, pT2N2aM0, Lt s/p MRI in MK 90
  According to the patient, She had regular follow up in GS OPD. And her CEA had slightly increased for a period of time. But the patient denied any GI symptoms.
  She was referred to GI OPD and recieved colonoscopy. It turned out to be Descending colon tumor. Therefore the patient was referred to CRS OPD.
  After discussion, the patient was admitted to our ward for surgical intervention.

體檢發現

-
  - General appearance :
    - easy looking , conscious clear , without cardiopulmonary distress
    - Vital signs自動帶入date 體溫(BT) 脈搏(P) 呼吸(R) 血壓(BP) 身高(BH) 體重(BW) BMI
      20200206 36℃ 83次/分 18次/分 109/61mmHg 159cm 44kg 17.4
  - HEENT :
    - Eyes: no anemic conjunctiva, no icteric sclera, normal EOM
    - ENT: no otorrhea, no rhinorrhea, no erythematous mucosa, no injected tonsil, no deformity
  - Neck: supple, freely movable, trachea midline,no deformity, no engorged jugular vein , no palpable thyroid or lymph node enlargement
  - Chest:
    - Inspection: Grossly normal, symmetric and fully expansion, no accessory muscle used, no operation scar, no spider angiomata, no gynecomastia
    - Palpation: no palpable mass or nodule, no subcutaneous emphysema, no tenderness
    - Ausculation: clear breathing sound
  - Heart:
    - Percussion: normal heart size
    - Ausculation: regular heart beat, normal S1, S2, no gallop, no murmur
  - Abdomen:
    - Inspection: in
      - flat contour, symetrically, no scar, no stria, no dilated veins, no rashes
    - Ausculation: normoactive bowel sound, no abdominal bruits
    - palpation: soft, no muscle guarding or rigidity, no local tenderness, no rebounding pain, no palpable mass, no palpable pulsation

- ○ Percussion: no shifting dulllness, no hepatomegaly, no splenomagaly
-

Peripheral pulsation :	CA	BA	RA	FA	PA	PTA	DPA
Right side :	++	++	++	++	++	++	++/+++
Left side :	++	++	++	++	++	++	++/+++

- Genito-anus: no tenderness or palpable mass lesion or rectal shelf, normal external genitalia, normal anal tone, brown stool

CRS CONDITION

-
  - ○ Lung

手術日期及方法

- 手術日期及方法20200207 Laparoscopic Anterior resection and anastomosis - malignant
  LEFT OOPHORECTOMY

住院治療經過

- After the admission, the patient received surgery on 2020/02/07 and tolerated it well. The patient recovered smoothly. Under relative stable condition, the patient was discharge on 2020/02/14.

合併症

- nil

一般檢查

- 累積報告 :

DATE	TIME	ALB	TP	BIL.T	BIL.D	ALKP	AST	ALT	LDH	CHOL	TG	HDL-C	LDL	UA	NH3	AMY
1090206	1920	4.7	7.4			59				201	77	55	118	3.7		
1090207	1415	3.8		1.1	0.2		23	6								
1090210	0937	3.1		0.67	0.19		14	4								

DATE	TIME	WBC	RBC	HGB	HCT	MCV	PLT	BLST	NEUT	BAND	SEG	LYM	MON	EOS	BAS
1090207	1419	11380	4.03	12.6	37.4		132		93.4			4.7	1.8	0.0	0.1
1090210	1007	7850	3.75	11.8	34.5	92.0	141		81.0			12.5	4.3	1.8	0.4

DATE	TIME	NA	K	CL	CA	BUN	CREAT	Mg	P	CRP	ESR	PCT	LIPASE	LACTATE
1090206	1920									0.042				
1090207	1415	140	4.3			9	0.64							
1090210	0859													6.4
1090210	0937	139	3.7			5	0.61							

DATE	TIME	GLUCOSE	PAB	TRF	ZN	CU	KETONE	CK	CKMB	PT-P	PT-C	APTT-P	APTT-C	TRO-T
1090206	1920	83												

特殊檢查

病理報告

其它

出院時情況

- 3.改門診治療

出院指示

-

藥名	劑量	劑量單位	用法	頻次	數量	數量單位	現況	天數	單位
Broen-C tab	1	TAB	PO	TID	9	TAB	TH	3	天
Voren-K tab 25mg	1	TAB	PO	TIDPRN	9	TAB	TH	3	天
Ultracet tab	1	TAB	PO	QID	12	TAB	TH	3	天
Ulstop tab 20mg	1	TAB	PO	BID	6	TAB	TH	3	天

出院計劃

主治醫師 2062F 蔣鋒帆 / 住院醫師 2328K 邱俊凱

正式摘要(電子簽章：**000000185808697)**

W83 / W83

doctorRank:B
